# CMR derived MAPSE and TAPSE Measurements in hypertrophic cardiomyopathy: comparison to healthy volunteers

**DOI:** 10.1186/1532-429X-14-S1-P168

**Published:** 2012-02-01

**Authors:** Hassan Abdel-Aty, Hugo A Katus, Stephanie Lehrke, Henning Steen

**Affiliations:** 1 Department of Cardiology, University of Heidelberg, Heidelberg, Germany; 2 Charité, Berlin, Germany

## Background

Mitral and tricuspid annular plane systolic excursion (MAPSE and TAPSE respectively) measurements are rapid, sensitive and reproducible means to assess left and right ventricular (LV, RV) function providing relevant prognostic information. Currently, only scarce data exists regarding the prognostic value of M/TAPSE in hypertrophic cardiomyopathy (HCM).

We hypothesized that M/TAPSE is reduced in HCM patients due to dysfunctional longitudinal contraction patterns and compared myocardial longitudinal function in HCM to a large cohort of healthy volunteers.

## Methods

**Figure 1:**
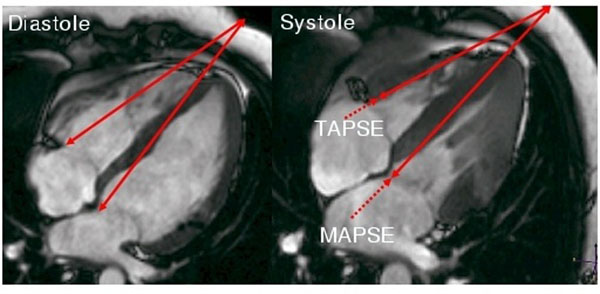


We studied 133 HCM patients (56±16 y, 94 men) and 120 healthy volunteers (42±13y, 60men) using 1.5T CMR scanner (Philips Achieva). Short axis slices covering the entire left ventricle were acquired using SSFP sequence to measure EF. MAPSE and TAPSE were measured in 4 chamber views. Comparable to echocardiography, two separate reference lines were drawn in diastole and systole from the basal lateral tricuspid (TAPSE) and the basal anterior mitral leaflet (MAPSE) to a reference point on the left chest surface (figure 1). P<0.05 was considered significant.

## Results

Both M/TAPSE were significantly higher in volunteers compared to HCM patients (MAPSE: 14±4 mm vs. 9±5 mm; p<0.0001 and TAPSE: 20±5 mm vs. 18±8 mm; p=0.002). Left ventricular EF was significantly higher in volunteers compared to HCM patients (65±9%; vs. 62±10% p=0.0003). When the entire population was considered (133 patients and 120 volunteers), there was a significant correlation between MAPSE and LVEF (r =0.24, p<0.0001). There were no significant gender related differences in M/TAPSE among HCM patients or healthy volunteers. M/TAPSE did not significantly correlate with LV mass in HCM patients and did not differ in HOCM (MAPSE: 9±4 mm, TAPSE: 19±8 mm) compared to HNCM (MAPSE: 8±4 mm, TAPSE: 18±7 mm; p=ns). ANOVA analysis showed no significant differences in TAPSE, MAPSE or EF based on patients’ NYHA class. Among patients, there was a significant inverse correlation between MAPSE and NT proBNP (r= -0.29, p=0.004) as well as between TAPSE and NT proBNP(r= -0.20, p=0.046).

## Conclusions

We provided reference values of M/TAPSE in a large cohort of HCM patients compared to a healthy study cohort. M/TAPSE were decreased in HCM compared to healthy volunteers and were not related to obstruction in HCM. The inverse relation to NT proBNP deserves further attention regarding a possible prognostic role of these measurements in HCM.

## Funding

None.

